# Pediatric Personalized Deep Learning Models for Segmentation of Hepatoblastoma at CT and MRI

**DOI:** 10.1148/rycan.250041

**Published:** 2026-02-20

**Authors:** Gourav Modanwal, Saurabh Kumar, Vidya Viswanathan, Cara E. Morin, Mitchell A. Rees, Judy H. Squires, Elizabeth R. Tang, Howard M. Katzenstein, Alexander J. Towbin, Anant Madabhushi, Gary R. Schooler

**Affiliations:** ^1^Wallace H. Coulter Department of Biomedical Engineering, Georgia Institute of Technology and Emory University, Health Sciences Research Building II, 1750 Haygood Dr, Ste N647, Atlanta, GA 30322; ^2^Department of Radiology, Cincinnati Children’s Hospital, Cincinnati, Ohio; ^3^Department of Radiology, University of Cincinnati College of Medicine, Cincinnati, Ohio; ^4^Department of Radiology, Nationwide Children’s Hospital, Columbus, Ohio; ^5^Department of Radiology, UPMC Children’s Hospital of Pittsburgh, Pittsburgh, Pa; ^6^Department of Radiology, Children’s Hospital of Colorado, Aurora, Colo; ^7^Nemours Children’s Hospital, Wilmington, Del; ^8^Atlanta Veterans Administration Medical Center, Atlanta, Ga

**Keywords:** Pediatrics, Deep Learning, Liver, MR-Imaging, Abdomen/GI, Algorithm Development

## Abstract

**Purpose:**

To evaluate the generalizability of adult-trained models for hepatoblastoma segmentation to pediatric patients and to develop two deep learning (DL) models, $M_{P}^{CT}$ and $M_{p}^{MRI}$, specifically trained on pediatric contrast-enhanced CT and T2-weighted MRI scans, respectively.

**Materials and Methods:**

Imaging data from the multicenter Children’s Oncology Group AHEP0731 trial (NCT00980460; May 2008–July 2018) were analyzed. DL models employing the three-dimensional U-Net architecture were trained using D_CT-Train_ and D_MRI-Train_. These models were evaluated on D_CT-Val_ and D_MRI-Val_ using the Dice similarity coefficient (DSC), and model segmentations were compared with manual segmentations from three annotators (R_1_, R_2_, and R_3_), their consensus (R_c_), and adult-trained model ($M_{A}^{CT}$) segmentations. Volume percentage error analysis was performed to evaluate segmentation precision.

**Results:**

A total of 104 participants (mean age ± SD, 28.2 months ± 30.5; 64 male; D_CT-Train_ = 56, D_CT-Val_ = 48) were included in the CT dataset and 123 (31.5 months ± 38.4; 87 male; D_MRI-Train _= 50, D_MRI-Val_ = 73) in the MRI dataset. $M_{P}^{CT}$ achieved good agreement with consensus segmentation (DSC = 0.86 [95% CI: 0.80, 0.91]) and exhibited higher agreement than $M_{A}^{CT}$with R_1_ (0.83 vs 0.55), R_2_ (0.85 vs 0.55), R_3_ (0.84 vs 0.54), and R_c _(0.86 vs 0.55) segmentations. Volume percentage error analysis revealed that $M_{P}^{CT}$ achieved segmentation results on par with or better than those of a novice annotator (R_3_) in high-precision scenarios. $M_{P}^{MRI}$ also achieved a DSC of 0.86, demonstrating good agreement with R_c_.

**Conclusion:**

The pediatric-trained DL-based models outperformed adult-trained models for accurate segmentation of pediatric hepatoblastoma.

**Keywords:** Pediatrics, Deep Learning, Liver, MR-Imaging, Abdomen/GI, Algorithm Development

ClinicalTrials.gov NCT00980460

*Supplemental material is available for this article.*

© The Author(s) 2026. Published by the Radiological Society of North America under a CC BY 4.0 license.

## Graphical abstract

**Figure:**
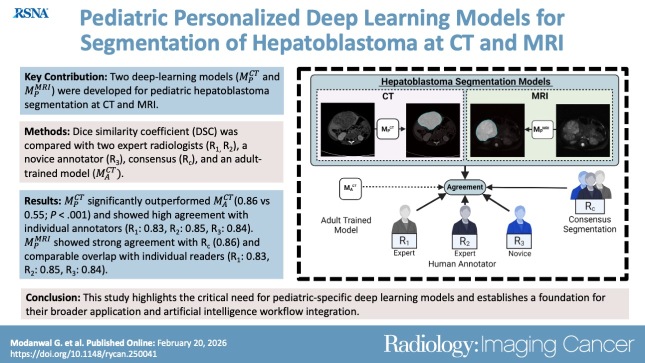

**Summary**

Deep learning–based hepatoblastoma segmentation models trained on pediatric CT and MRI scans ($M_{P}^{CT}$ and $M_{P}^{MRI}$) demonstrated good agreement with human annotators and their consensus. $M_{P}^{CT}$ outperformed the adult CT-trained model ($M_{A}^{CT}$).

**Key Points**

■ Two deep learning–based hepatoblastoma segmentation models, $M_{P}^{CT}$ and $M_{P}^{MRI}$, were trained on pediatric contrast-enhanced CT and T2-weighted MRI scans, respectively.
■ $M_{P}^{CT}$ demonstrated strong agreement with two expert and one novice human annotator and their consensus (R_c_) segmentation (Dice similarity coefficients: 0.83, 0.85, 0.84, and 0.86, respectively);$M_{P}^{MRI}$ also demonstrated good agreement with R_c_ (DSC = 0.86).
■ Agreement between $M_{P}^{CT}$ and R_c_ (DSC = 0.86) was greater than that between R_c_ and an adult CT-trained model (DSC = 0.55) (*P* ≤ .0001).

## Introduction

Hepatoblastoma is the most common hepatic malignancy of childhood, yet it is a rare disease with an incidence of 2.3 cases per million children per year (1). The rarity of the disease is compounded by its different pathologic types and subtypes, and because it occasionally occurs in patients with underlying liver or genetic diseases. Most commonly hepatoblastoma has been categorized pathologically as either epithelial or mixed epithelial-mesenchymal (2). However, as our understanding of the disease has grown, the number of histologic subtypes has expanded. Currently, pathologists recognize nine histologic subtypes of epithelial hepatoblastoma and two types of mixed epithelial-mesenchymal hepatoblastoma (3). Identification of some tumor subtypes may help guide therapeutic decision-making (4,5). Currently, hepatoblastoma pathologic subtype analysis of tumors relies on histologic assessment often occurring in limited tumor tissue obtained at biopsy at diagnosis (6). However, a few radiomics tools (7,8) have shown potential in identifying distinct tumor subtypes through imaging analysis, which may complement biopsy in ascertaining the diagnosis and better predict tumor heterogeneity. Radiomics has the potential to increase diagnostic accuracy, improve disease monitoring, and optimize treatment planning (9).

The first step of any radiomics analysis (10,11) is to identify regions of interest in the images for subsequent analysis using artificial intelligence (AI) frameworks. Segmenting these regions of interest, especially for complex lesions such as liver tumors, has traditionally been a time-consuming process that requires substantial manual input and a high level of subject matter expertise (12). Differences in subject matter expertise among manual human annotators can lead to interrater variability (13), which can introduce potential inconsistencies (14) in the AI analysis. Automating this segmentation task using AI can improve efficiency in the image analysis workflow and reduce variability in tumor segmentations.

**Figure 1:**
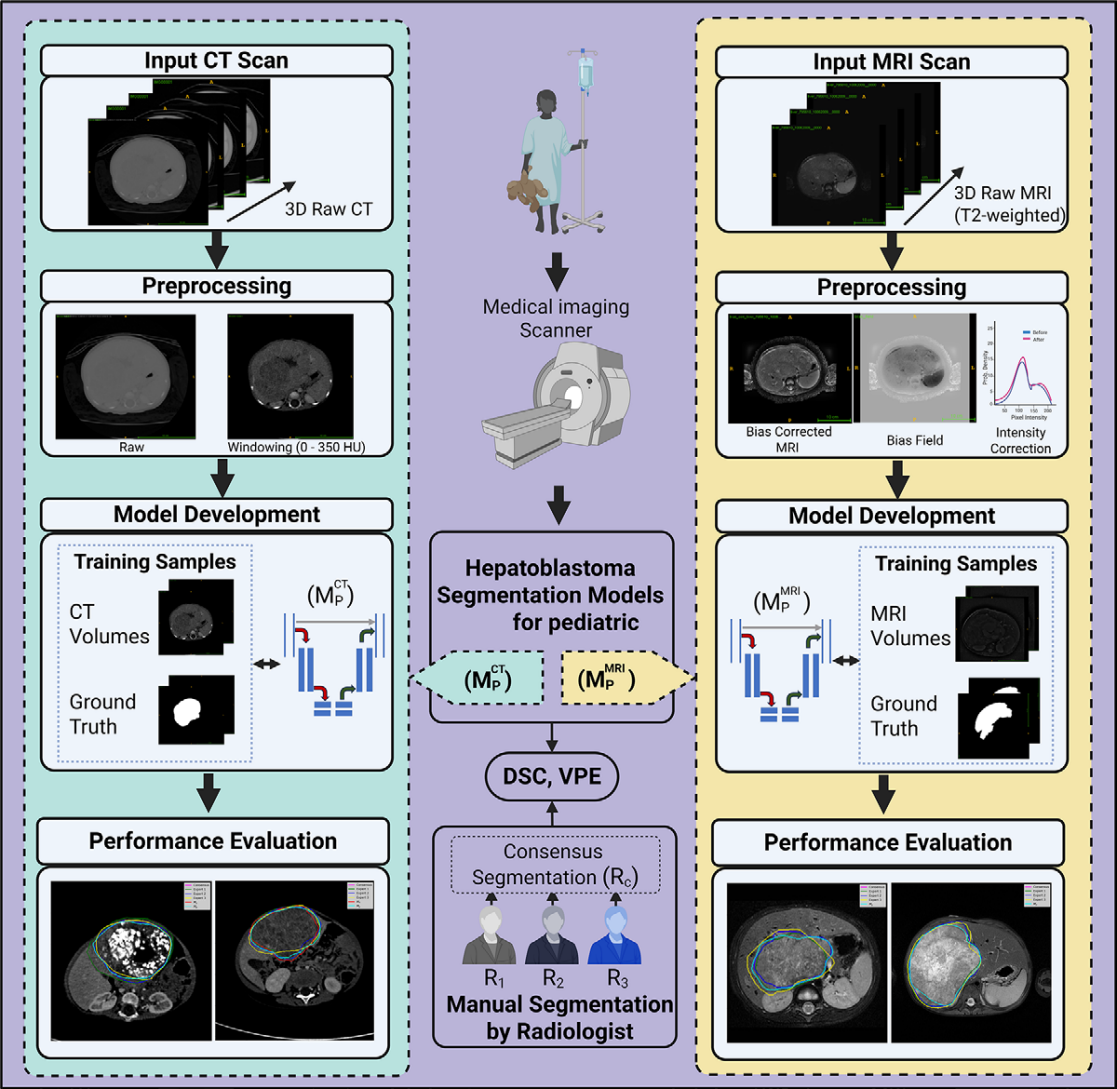
Image shows an overview of the study workflow for developing and evaluating deep learning models trained on pediatric contrast-enhanced CT images ($M_{P}^{CT}$; left) and T2-weighted MRI scans ($M_{P}^{MRI}$; right) for pediatric hepatoblastoma segmentation. Both segmentation models were trained using the nnU-Net framework. The models were evaluated by calculating the Dice similarity coefficient (DSC) and compared with manual segmentations from three annotators (R_1_, R_2_, and R_3_) and their consensus (R_c_). Additionally, volume percentage error (VPE) analysis was conducted to assess segmentation performance. 3D = three-dimensional.

The field of AI has rapidly expanded its applications in radiology, offering potential for improved diagnosis and prognosis (15). However, a concerning disparity exists in pediatric cancer imaging. Most of the research efforts have predominantly concentrated on adults (16), leaving the pediatric population underrepresented and resulting in inequitable access to these technologies (17). For adults, multiple techniques have been described, including the employment of deep learning (DL) methods for liver and liver tumor segmentation (16,18). These models have not been validated in children, and evidence suggests that machine learning models trained on data from adult samples may not necessarily translate effectively to the pediatric population (19–21). To our knowledge, there is no empirical evidence yet on whether adult-trained models perform adequately for pediatric liver tumor segmentation. In this work, we hypothesized that models developed using pediatric data would perform similar to human annotators and outperform an adult-based model. Therefore, we aimed to evaluate the generalizability of adult-trained models and develop two DL-based segmentation models explicitly tailored for pediatric patients—one trained on contrast-enhanced CT scans ($M_{P}^{CT}$) and another on T2-weighted MRI scans ($M_{P}^{MRI}$). We further compared their performance with human annotators and quantified segmentation precision and bias using volume percentage error (VPE) analysis. An overview of the study’s workflow is illustrated in Figure 1.

## Materials and Methods

This secondary analysis used de-identified imaging from the multicenter, prospective Children’s Oncology Group AHEP0731 clinical trial (NCT00980460; May 2008–July 2018) (22). Institutional review board approval and informed consent were obtained at participating sites for the parent trial; the present analysis of de-identified data required no additional review or exemption per local policy and was conducted in accordance with the Health Insurance Portability and Accountability Act.

This study was supported in part by funding from the St. Baldrick’s Foundation and the National Cancer Institute under multiple award numbers. This study did not receive direct support from any industry or commercial organization specific to the research described. All authors had full control over the data collection, analysis, interpretation, and the decision to submit the manuscript for publication. Although some authors have industry affiliations, none of these relationships influenced the conduct or reporting of this study.

### Study Dataset

The imaging dataset included 226 unique participants with 358 CT scans and 144 MRI scans acquired at prespecified study time points at 105 unique health care centers in the United States and Canada. A portion of this anonymized dataset is publicly available via The Cancer Imaging Archive *(https://www.cancerimagingarchive.net/collection/ahep0731/)*. A primary study objective was the use of imaging in guiding surgical planning, particularly through the use of the pretreatment extent of tumor (PRETEXT) staging system. Some participants included in this analysis have been reported previously (4), which focused on clinical outcomes of the AHEP0731 trial. The current study focuses exclusively on the development and validation of DL-based hepatoblastoma segmentation models. PRETEXT classifies liver tumor extent before treatment based on the number of uninvolved liver sections. The liver is divided into four anatomic sections—left lateral, left medial, right anterior, and right posterior—based on Couinaud segmentation. PRETEXT groups range from I (three uninvolved sections) to IV (all four sections involved), and this system is routinely used in pediatric liver cancer trials to inform treatment and surgical strategies. PRETEXT staging data were available for all cases in this cohort.

We randomly selected participants from a convenience sample of AHEP0731 enrollees who met all of the following: pathologically confirmed hepatoblastoma and baseline abdominal imaging at diagnosis (portal venous phase contrast-enhanced CT or T2-weighted fat-suppressed MRI). Participants who did not meet each of these criteria were not eligible for inclusion. No further exclusion criteria were applied beyond those established in the original trial protocol.

The CT dataset (D_CT_, *n* = 104) was split into a training set (D_CT-Train_, *n* = 56) and a validation set (D_CT-Val_, *n* = 48), both consisting of randomly selected portal venous phase CT scans for the development and evaluation of the $M_{P}^{CT}$ model. Similarly, the MRI dataset (D_MRI_, *n* = 123) was also split into a training set (D_MRI-Train_, *n* = 50) and a validation set (D_MRI-Val_, *n* = 73), both consisting of T2-weighted fat-suppressed MRI series, and these sets were used for the development and evaluation of the $M_{P}^{MRI}$ model. D_CT-Val _and D_MRI-Val _were held-out evaluation sets used only for final performance estimation; no hyperparameter tuning was performed on these sets. All imaging was considered standard of care, and locally defined imaging protocols were used.

### Manual Segmentation by Human Annotators

Three annotators (R_1_ [A.J.T.], R_2_ [G.R.S.], and R_3_ [V.V.]) independently performed manual segmentation on tumors blinded to each other’s segmentation results. R_1_ and R_2_, both board-certified pediatric radiologists with a certificate of added qualification, had 17 and 14 years of experience, respectively. Both R_1_ and R_2_ had expertise in pediatric liver tumor imaging and had served as expert radiology reviewers for Children’s Oncology Group Liver Tumor Trials. R_3_ was considered a novice. R_3_ was a recent medical school graduate in radiology training at the time of study, serving as an imaging research fellow. Manual segmentation was performed using ITK-SNAP version 4.0 (Penn Image Computing & Science Laboratory) and 3D Slicer version 5.8.1 (Brigham and Women’s Hospital).

For both CT and MRI datasets, the training sets (D_CT-Train_ and D_MRI-Train_) were annotated by R_2_, who manually constructed three-dimensional tumor segmentations for all imaging examinations. In contrast, for the validation sets (D_CT-Val_ and D_MRI-Val_), manual three-dimensional tumor segmentations were independently generated by all the three annotators (R_1_, R_2_, and R_3_). Consensus segmentation (R_c_) was obtained by combining the manual segmentations from three annotators, where a pixel was labeled as part of the tumor if at least two of the three annotators agreed. This majority agreement at the pixel level helped establish R_c_. This approach reduces individual biases and variability, resulting in a more accurate and consistent reference standard segmentation; offers increased reliability over individual annotators; and serves as a strong benchmark for evaluating DL models.

**Data preparation**

Images were provided as Digital Imaging and Communications in Medicine files and were converted to the Neuroimaging Informatics Technology Initiative format for preprocessing, training, and evaluation. All images were resampled to a consistent voxel spacing (median of training set) using third-order spline interpolation for image data and nearest-neighbor interpolation for segmentation masks. Preprocessing was performed to standardize image intensities and improve generalizability across imaging sites. This step helps to make the analysis pipeline generalizable to all images within and outside of the dataset. For CT, we confined the pixel intensities to a soft tissue window of 0–350 HU (23). For MRI, we first performed bias correction (24) as an initial step to reduce the low-frequency, multiplicative bias field, which can cause uneven intensity distributions across the image. This step is essential for improving the uniformity of tissue signal intensities, thereby improving the accuracy of subsequent image analyses. Furthermore, given the qualitative nature of MRI, we standardized the liver region (including the tumor) in our images with respect to a template image and liver mask (25). To create a liver mask for standardization, we trained an nnU-Net model on 20 three-dimensional images of the liver and the corresponding mask from the Combined Healthy Abdominal Organ Segmentation dataset (26). Using the nnU-Net model trained on the Combined Healthy Abdominal Organ Segmentation dataset, we then generated a segmentation of the liver region in our dataset. Using the segmented liver and tumor regions, we applied histogram standardization techniques (25) to normalize the MRI scans based on the template image and mask within our dataset. Inference-time ablation results evaluating the contribution of individual preprocessing steps are provided in Table S1.

**DL models development and evaluation**

**Figure 2:**
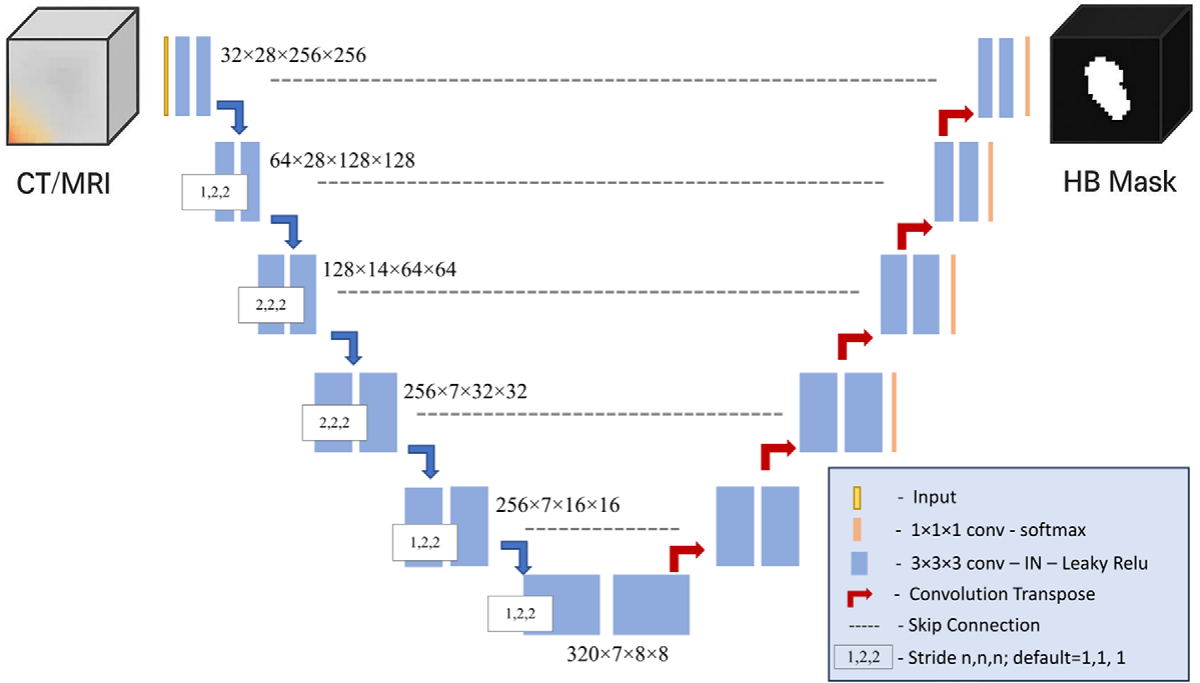
Schematic of the three-dimensional U-Net architecture used for tumor segmentation. The network adopts an encoder-decoder design with skip connections that preserve spatial context. Each block consists of 3 × 3 × 3 convolutions followed by instance normalization (IN) and leaky ReLU (rectified linear unit) activation. Downsampling is performed using strided convolutions, and upsampling is achieved through transposed convolutions. Skip connections from the encoder to decoder help retain fine-grained details. The final layer applies a 1 × 1 × 1 convolution with softmax to generate voxelwise class probabilities. HB mask = hepatoblastoma tumor segmentation.

The model architecture followed the standard three-dimensional U-Net configuration as implemented in the nnU-Net framework (27). It employs a symmetric five-stage encoder-decoder structure with long-range skip connections that preserve spatial context across resolution levels (Fig 2). Each stage comprises two 3 × 3 × 3 convolutions followed by instance normalization and leaky rectified linear unit activation, supporting stable training and efficient feature extraction. Downsampling and upsampling are performed using strided and transposed convolutions, respectively, enabling hierarchical feature learning. Channel depth increases progressively from 32 to 320, enhancing the network’s capacity to model complex tumor morphologies. At the bottleneck, anisotropic kernel configurations (1 × 2 × 2) are used to account for the lower through-plane resolution. The three-dimensional U-Net architecture was trained de novo (ie, without any pretraining) to segment the tumor using nnU-Net framework. After preprocessing, we trained the models using the training set. The generalized Dice loss (28) was used as the optimization objective because it accounts for class imbalance by weighting classes inversely proportional to their volume. We set the initial learning rate to 1× 10^−2^ and applied a weight decay of 3 × 10^−5^. The learning rate was reduced on a plateau when validation performance ceased to improve, ensuring the model converged effectively while minimizing unnecessary training. Standard augmentation, including spatial transformations (random rotations up to ±30°, scaling within 0.7–1.4×, elastic deformations), intensity-based adjustments (gamma correction, brightness augmentation), and mirroring across axes, were applied. A total of 5000 epochs were used for $M_{P}^{CT}$ and 1000 epochs for $M_{P}^{MRI}$. Additionally, for CT scans only, we had the ability to compare performance of $M_{P}^{CT}$ to $M_{A}^{CT}$, a publicly available liver tumor segmentation model trained on adult data (29).

### Statistical Analysis

We used the Dice similarity coefficient (DSC), calculated as the spatial overlap between binary segmentation masks (1 = tumor, 0 = nontumor) from the automated and reference standard tumor segmentations, to assess agreement: greater than 0.9 (excellent), 0.80–0.89 (good), 0.70–0.79 (fair), and less than 0.70 (poor). DSC was computed at the image level, and each image contributed equally. Beyond evaluating DSC agreement with the reference standard segmentation, we aimed to better understand the model’s performance and potential segmentation precision and bias by analyzing predicted volume (PV) and true volume (TV) obtained with respect to R_c_. We acknowledge that models, like human decision-making, are prone to errors, but certain levels of inaccuracy may be acceptable depending on the specific task or application (30). By evaluating accuracy within acceptable limits defined by error tolerance thresholds, we can gauge model reliability and suitability for practical use in different tasks. We conducted a VPE analysis to assess accuracy across various error tolerance thresholds (very low: 1%, low: 5%, moderate: 10%, and high: 15%). Specifically, we measured the percentage of cases where the model’s volume error (e) (31) fell within predefined error tolerance thresholds (eg, e < 1%, e < 5%, etc), assessing how often the predictions and annotations stayed within acceptable limits. This analysis provides a quantitative measure of the ability of models to estimate tumor volumes accurately and is essential for understanding the clinical applicability of the models, where precise volume estimation is critical for reliable outcomes.

Additionally, we also analyzed the PV/TV ratio to understand model tendency toward undersegmentation (missed tumor regions) or oversegmentation (including irrelevant background pixels). The consensus segmentation (R_c_), derived from majority agreement among three annotators, served as the reference standard for TV estimation. The percentage of cases with PV/TV ratios greater than 1 indicated potential oversegmentation, whereas those with ratios less than or equal to 1 suggested undersegmentation. The median PV/TV ratio provided an overall measure of segmentation tendency. Statistical significance was determined using the one-sample Wilcoxon signed rank test, which highlighted any significant differences from unity.

We estimated voxelwise model uncertainty by computing the Shannon entropy of the softmax output at inference. Higher entropy values correspond to greater predictive uncertainty. Entropy maps were generated for qualitative assessment, and mean entropy values were calculated per case to explore the relationship between uncertainty and segmentation performance. Additionally, we computed mean entropy within two tumor subregions: the core, defined as the eroded interior of the tumor mask using a two-dimensional disc-shaped structuring element (radius = 10 pixels), and the boundary, defined as the outer rim of the tumor mask (original tumor mask minus the eroded core), capturing edge regions prone to segmentation uncertainty. These subregion-level metrics allowed us to assess spatial patterns of uncertainty.

We used the Python (version 3.10.3; *https://www.python.org/downloads/release/python-3103/*), nnU-Net (version 1.7.1; *https://github.com/MIC-DKFZ/nnUNet*), PyTorch (version 1.10; *https://pytorch.org/get-started/previous-versions/*), SimpleITK (version 2.4.0; *https://simpleitk.readthedocs.io/en/v2.4.0/gettingStarted.html*), and SciPy (version 1.10.1; *https://docs.scipy.org/doc/scipy-1.10.1/*) packages for preprocessing, model training, and statistical analyses. All reported *P* values were two-sided, and a *P* value less than .05 was considered statistically significant. 95% CIs were computed using standard normal approximation methods.

## Results

### Dataset Characteristics

The participant characteristics for the CT datasets (D_CT-Train_ and D_CT-Val_) and the MRI datasets (D_MRI-Train_ and D_MRI-Val_) are summarized in Table 1. For the CT datasets, the 104 participants (40 female, 64 male) had a mean age of 28.2 months ± 30.5 (range, 2.0–170.0 months). PRETEXT grouping was as follows: two of 104 (1.9%) in group I, 30 of 104 (28.8%) in group II, 45 of 104 (43.3%) in group III, and 27 of 104 (26.0%) in group IV. For the MRI datasets, the 123 participants (35 female, 87 male, one missing) had a mean age of 31.5 months ± 38.4 (range, 0.0–189.0 months). PRETEXT distribution in the MRI cohort was group I, five of 123 (4.1%); group II, 36 of 123 (29.5%); group III, 64 of 123 (52.5%); and group IV, 17 of 123 (13.9%).

**Table 1:** Summary of Participant Characteristics for the CT and MRI Datasets

Characteristic	CT			MRI		
	Overall (*n* = 104)	Training Set (*n* = 56)	Validation Set (*n* = 48)	Overall (*n* = 123)	Training Set (*n* = 50)	Validation Set (*n* = 73)
Age (mo)*	28.2 **±** 30.5	32.1 **±** 35.4	23.7 **±** 23.1	31.5 **±** 38.4	31.5 **±** 41.4	31.6 **±** 36.5
Sex
Female*	40 (38.5)	23 (41.1)	17 (35.4)	35 (28.7)	16 (32.0)	19 (26.4)
Male	64 (61.5)	33 (58.9)	31 (64.6)	87 (71.3)	34 (68.0)	53 (73.6)
PRETEXT grouping						
Group I*	2 (1.9)	1 (1.8)	1 (2.1)	5 (4.1)	3 (6.0)	2 (2.8)
Group II	30 (28.8)	11 (19.6)	19 (39.6)	36 (29.5)	13 (26.0)	23 (31.9)
Group III	45 (43.3)	29 (51.8)	16 (33.3)	64 (52.5)	26 (52.0)	38 (52.8)
Group IV	27 (26.0)	15 (26.8)	12 (25.0)	17 (13.9)	8 (16.0)	9 (12.5)

Note.—Data are presented as means ± SDs or numbers with percentages in parentheses. PRETEXT = pretreatment extent of disease.

* Data are missing from one MRI case.

**Figure 3:**
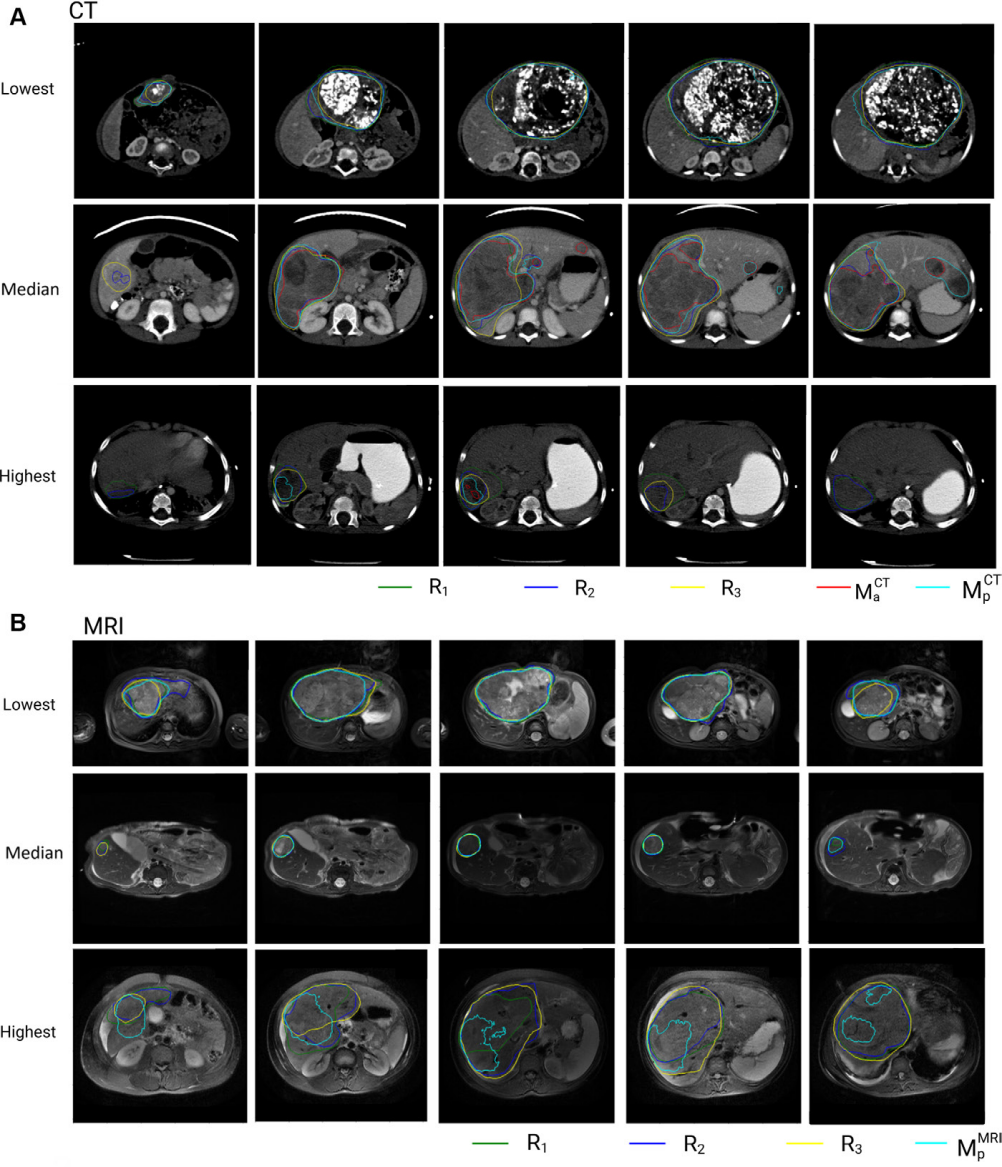
Example images of hepatoblastoma segmentation for the cases with the best, median, and worst DSC score on **(A)** CT and **(B)** MRI scans.

Examples of the DL-based automated segmentations ($M_{P}^{CT}$, $M_{A}^{CT}$, and $M_{P}^{MRI}$) and manual segmentations (R_1_, R_2_, R_3_) are shown in Figure 3. This figure highlights cases where $M_{P}^{CT}$ achieves the highest, median, and lowest DSC scores compared with R_c_. Interrater DSC agreement among reference standard (ie, annotators [R_1_, R_2_, R_3_]), consensus segmentation (ie, R_c_), and DL-based models (ie, $M_{P}^{CT}$ and $M_{A}^{CT}$) is presented in Table 2. The corresponding results for MRI segmentations ($M_{P}^{MRI}$) are shown in Table 3.

**Table 2:** Interrater Agreement between Annotators’ Manual Assessments and Deep Learning Model Segmentations on CT Images

	R_c_(Consensus)	R_1_ (Expert)	R_2_ (Expert)	R_3_ (Novice)	$M_{P}^{CT}$	$M_{A}^{CT}$
R_c _(consensus)	1	0.94 (0.93, 0.96)	0.95 (0.94, 0.96)	0.94 (0.92, 0.95)	0.86 (0.80, 0.91)	0.55 (0.46, 0.65)
R_1_ (expert)	0.94 (0.93, 0.96)	1	0.89 (0.87, 0.91)	0.88 (0.85, 0.90)	0.83 (0.78, 0.89)	0.55 (0.46, 0.64)
R_2_ (expert)	0.95 (0.94, 0.96)	0.89 (0.87, 0.91)	1	0.88 (0.86, 0.90)	0.85 (0.79, 0.90)	0.55 (0.46, 0.64)
R_3_ (novice)	0.94 (0.92, 0.95)	0.88 (0.85, 0.90)	0.88 (0.86, 0.90)	1	0.84 (0.79, 0.89)	0.54 (0.45, 0.65)
$M_{P}^{CT}$	0.86 (0.80, 0.91)	0.83 (0.78, 0.89)	0.85 (0.79, 0.90)	0.84 (0.79, 0.89)	1	0.58 (0.48, 0.67)
$M_{A}^{CT}$	0.55 (0.46, 0.65)	0.55 (0.46, 0.64)	0.55 (0.46, 0.64)	0.54 (0.45, 0.65)	0.58 (0.48, 0.67)	1

Note.—Data are presented as Dice similarity coefficients with 95% CIs in parentheses. $M_{P}^{CT}$ and $M_{P}^{MRI}$ are the models trained on pediatric contrast-enhanced CT and T2-weighted MRI scans, respectively; $M_{A}^{CT}$ is a publicly available liver tumor segmentation model trained on adult CT data.

**Table 3:** Interrater Agreement between Annotators’ Manual Assessments and Deep Learning Model Segmentations on MRI Scans

	R_c_ (Consensus)	R_1_ (Expert)	R_2_ (Expert)	R_3_ (Novice)	$M_{P}^{MRI}$
R_c_ (consensus)	1	0.95 (0.94, 0.96)	0.95 (0.94, 0.96)	0.94 (0.92, 0.95)	0.86 (0.80, 0.91)
R_1_ (expert)	0.95 (0.94, 0.96)	1	0.90 (0.88, 0.91)	0.88 (0.86, 0.90)	0.83 (0.78, 0.87)
R_2_ (expert)	0.95 (0.94, 0.96)	0.90 (0.88, 0.91)	1	0.88 (0.86, 0.90)	0.85 (0.79, 0.90)
R_3_ (novice)	0.94 (0.92, 0.95)	0.88 (0.86, 0.90)	0.88 (0.86, 0.90)	1	0.84 (0.79, 0.89)
$M_{P}^{MRI}$	0.86 (0.80, 0.91)	0.83 (0.78, 0.87)	0.85 (0.79, 0.90)	0.84 (0.79, 0.89)	1

Note.—Data are presented as Dice similarity coefficients with 95% CIs in parentheses. $M_{P}^{MRI}$ is a deep learning model trained on pediatric T2-weighted MRI scans.

### CT Model Performance and VPE Analysis

The agreement between $M_{P}^{CT}$ and R_c_ was good, with a DSC of 0.86 (95% CI: 0.80, 0.91). Similarly, $M_{P}^{CT}$ exhibited good agreement with individual annotators, achieving a DSC of 0.83 (95% CI: 0.78, 0.89) with R_1_, 0.85 (95% CI: 0.79, 0.90) with R_2_, and 0.84 (95% CI: 0.79, 0.89) with R_3_. In comparison to $M_{A}^{CT}$, $M_{P}^{CT}$ consistently achieved higher DSCs across all annotator comparisons. Specifically, the DSC for agreement between $M_{P}^{CT}$ and R_c_, 0.86 (95% CI: 0.80, 0.91), was significantly (*P* < .0001) greater than that for agreement between $M_{A}^{CT}$ and R_c_ (0.55; 95% CI: 0.46, 0.65). Consistent differences were observed in the DSC agreement with R_1_ ($M_{P}^{CT}$: 0.83 vs $M_{A}^{CT}$: 0.55; *P* < .001), R_2_ ($M_{P}^{CT}$: 0.85 vs $M_{A}^{CT}$: 0.55; *P* < .001), and R_3_ ($M_{P}^{CT}$: 0.84 vs $M_{A}^{CT}$: 0.54; *P* < .001). Additionally, the DSC for agreement between $M_{A}^{CT}$ and $M_{P}^{CT}$ was 0.58 (95% CI: 0.48, 0.67), indicating poor agreement.

The VPE analysis presented in Table 4 reveals that the expert annotators (R_1_ and R_2_) generally achieved higher accuracy than the novice annotator (R_3_) and the DL models ($M_{A}^{CT}$ and $M_{P}^{CT}$) across all predefined error tolerances. Specifically, R_1_ excelled at very low error tolerance threshold (ie, very precise segmentation), achieving segmentation within 1% error for seven of 48 (14.58%) cases and within 5% error for 33 of 48 (68.75%) cases. R_2_ and R_3_ demonstrated better performance at high error tolerance threshold (15%), with R_2_ achieving segmentation within 15% error for 44 of 48 (91.67%) cases and R_3_ for 43 of 48 (89.58%) cases. Among the DL models, $M_{P}^{CT}$ matched or outperformed the novice annotator (R_3_) in precise segmentation, achieving segmentation within 1% error for five of 48 (10.42%) cases and within 5% error for 28 of 48 (58.33%) cases, compared with R_3_’s 26 of 48 (54.17%). $M_{P}^{CT}$ demonstrated a reasonable performance, particularly at moderate (e < 10%: 36 of 48 [75%]) and high (e < 15%: 40 of 48 [83.33%]) tolerance threshold, but $M_{A}^{CT}$ underperformed across all error tolerance thresholds.

**Table 4:** Volume Percentage Error Analysis of Liver Tumor Segmentation Models Trained on CT and MRI Scans

Modality	Error Tolerance	R_1_ (Expert)	R_2_ (Expert)	R_3_ (Novice)	$M_{A}^{CT}$	$M_{P}^{CT}$ or$M_{P}^{MRI}$
CT (*n* = 48)	Very low (e < 1)	7 (14.58)	5 (10.42)	5 (10.42)	0 (0.00)	5 (10.42)
	Low (e < 5)	33 (68.75)	33 (68.75)	26 (54.17)	0 (0.00)	28 (58.33)
	Moderate (e < 10)	40 (83.33)	38 (79.17)	40 (83.33)	1 (2.08)	36 (75.00)
	High (e < 15)	42 (87.50)	44 (91.67)	43 (89.58)	4 (8.33)	40 (83.33)
MRI (*n* = 73)	Very low (e < 1)	15 (20.55)	8 (10.96)	7 (9.59)	NA	6 (8.22)
	Low (e < 5)	50 (68.49)	52 (71.23)	43 (58.90)	NA	28 (38.36)
	Moderate (e < 10)	63 (86.30)	62 (84.93)	58 (79.45)	NA	45 (61.64)
	High (e < 15)	68 (93.15)	67 (91.78)	64 (87.67)	NA	53 (72.60)

Note.—Data are numbers with percentages in parentheses. The error tolerance levels and corresponding accuracy for expert annotators (R_1_, R_2_), a novice annotator (R_3_), and deep learning models ($M_{A}^{CT}$ and $M_{P}^{CT}$ or$M_{P}^{MRI}$) compared with consensus segmentation. Accuracy is defined as the percentage of cases where segmentation errors are below specified error tolerance levels (e < 1, e < 5, e < 10, e < 15). $M_{P}^{CT}$ and $M_{P}^{MRI}$ are models trained on pediatric contrast-enhanced CT and T2-weighted MRI scans, respectively; $M_{A}^{CT}$ is a publicly available liver tumor segmentation model trained on adult CT data. NA = not applicable.

### MRI Model Performance and VPE Analysis

DSCs for MRI segmentations are presented in Table 3. $M_{P}^{MRI}$ demonstrated good agreement with different levels of expertise and their consensus. The DSC for agreement between $M_{P}^{MRI}$ and R_c_ was 0.86 (95% CI: 0.80, 0.91). In comparisons of individual annotators, the DSCs were as follows: 0.83 (95% CI: 0.78, 0.87) for R_1_, 0.85 (95% CI: 0.79, 0.90) for R_2_, and 0.84 (95% CI: 0.79, 0.89) for R_3_.

The VPE analysis (refer to Table 4) demonstrated that the expert annotators (R_1_ and R_2_) consistently achieved superior accuracy across all predefined error tolerance thresholds when compared with the novice annotator (R_3_) and the DL model ($M_{P}^{MRI}$). Specifically, R_1_ segmented within 1% error for 15 of 73 (20.55%) cases and within 5% error for 50 of 73 (68.49%) cases. R_2_ achieved comparable segmentation performance within 1% error for eight of 73 (10.96%) cases and within 5% error for 52 of 73 (71.23%) cases. Both experts maintained high accuracy at higher error margins, with R_1_ segmenting within 15% error for 68 of 73 (93.15%) cases and R_2_ for 67 of 73 (91.78%) cases. In comparison, the novice annotator (R_3_) had slightly lower accuracy, segmenting within 1% error for seven of 73 (9.59%) cases and within 5% error for 43 of 73 (58.90%) cases. The DL model ($M_{P}^{MRI}$) performed closely to R_3_, segmenting within 1% error for six of 73 (8.22%) cases and within 5% error for 28 of 73 (38.36%) cases, indicating a notable drop in accuracy compared with the experts. However, at the moderate (10%) and high (15%) tolerance thresholds, $M_{P}^{MRI}$ showed moderate performance, with 45 of 73 (61.64%) and 53 of 73 (72.60%) cases falling within these errors, respectively, though the overall accuracy of the DL model ($M_{P}^{MRI}$) was less than that of both expert annotators and novice annotator R_3_.

### Under- and Oversegmentation Tendencies

Trends toward under- and oversegmentation relative to R_c_ are summarized in Table 5. In the CT analysis, annotators showed a mix of under- and oversegmentation: R_1_ leaned toward undersegmentation, with 31 of 48 (64.6%) cases having a PV/TV less than or equal to 1, whereas R_2_ and R_3_ exhibited slight oversegmentation, with 32 of 48 (66.7%) and 26 of 48 (54.2%) cases having a PV/TV greater than 1; the median PV/TV was 1.02 and 1.01, respectively. By contrast, the DL models, particularly $M_{A}^{CT}$, demonstrated a marked tendency toward severe undersegmentation: only one of 48 (2.1%) cases had a PV/TV greater than 1, with a median PV/TV of 0.56; this shift toward undersegmentation was statistically significant (*P* < .05). The undersegmentation tendency persisted for $M_{P}^{CT}$, though less pronounced (median PV/TV = 0.97). For MRI, R_1_ and R_3_ leaned toward oversegmentation—38 of 73 (52.1%) cases and 46 of 73 (63.0%) cases with a PV/TV greater than 1—whereas R_2_ tended toward undersegmentation—40 of 73 (54.8%) cases with a PV/TV less than or equal to 1. The DL model $M_{P}^{MRI}$ showed a modest undersegmentation tendency, with 44 of 73 (60.3%) cases having a PV/TV less than or equal to 1 and a median PV/TV of 0.98.

**Table 5:** Analysis of Under- and Oversegmentation Tendency

Modality	Metric	Expert 1	Expert 2	Expert 3	$M_{A}^{CT}$	$M_{P}^{CT}$ or$M_{P}^{MRI}$
CT (*n* = 48)	PV/TV > 1	17 (35.42)	32 (66.67)	26 (54.17)	1 (2.08)	15 (31.25)
	PV/TV ≤ 1	31 (64.58)	16 (33.33)	22 (45.83)	47 (97.92)	33 (68.75)
	Median PV/TV	0.99	1.02	1.01	0.56	0.97
	*P* value	.1066	.2684	.947	4.97 × 10^−14^	.0021
	Tendency	Undersegmentation	Oversegmentation	Oversegmentation	Undersegmentation	Undersegmentation
MRI (*n* = 73)	PV/TV > 1	38 (52.05)	33 (45.21)	46 (63.01)	—	29 (39.73)
	PV/TV ≤ 1	35 (47.95)	40 (54.79)	27 (36.99)	—	44 (60.27)
	Median PV/TV	1	0.99	1.02	—	0.98
	*P* value	.33	.8452	.1312	—	.0526
	Tendency	Oversegmentation	Undersegmentation	Oversegmentation	—	Undersegmentation

Note.—Data are numbers with percentages in parentheses for PV/TV > 1 and PV/TV ≤ 1. The statistical significance of differences between PV and TV was assessed using the one-sample WSRT. $M_{P}^{CT}$ and $M_{P}^{MRI}$ are models trained on pediatric contrast-enhanced CT and T2-weighted MRI scans, respectively; $M_{A}^{CT}$ is a publicly available liver tumor segmentation model trained on adult CT data. PV = predicted volume, TV = true volume, WSRT = Wilcoxon signed rank test.

### Entropy-based Uncertainty Analysis

Entropy analysis revealed that uncertainty was primarily concentrated along tumor boundaries (Fig S1). Quantitatively, mean entropy in the boundary region was higher than in the core across both imaging modalities (Fig S2). For CT, the median of the mean entropy across test cases was 0.05 in the core (IQR, 0.02–0.08) and 0.27 in the boundary region (IQR, 0.23–0.30). For MRI, the corresponding values were 0.01 for the core (IQR, 0.002–0.039) and 0.13 for the boundary (IQR, 0.09–0.16).

## Discussion

Accurate segmentation of pediatric liver tumors is a critical first step in many analyses, including those employing AI techniques for effective diagnosis, treatment planning, and monitoring for treatment response (10). However, most existing AI research (16,18) has focused predominantly on adult samples. Because of the distinct anatomic and pathologic characteristics of pediatric patients, adult-trained models have failed to generalize effectively to this population. In this work, we developed two segmentation models, $M_{P}^{CT}$ and $M_{P}^{MRI}$, tailored for pediatric hepatoblastoma. $M_{P}^{CT}$ was trained on contrast-enhanced CT images, and $M_{P}^{MRI}$ was trained on T2-weighted fat-suppressed MRI scans, both sourced from the Children’s Oncology Group AHEP0731 trial. We evaluated these models in terms of their ability to provide efficient and reliable segmentation solutions for pediatric hepatoblastomas. Furthermore, we conducted a VPE analysis to assess the precision of tumor segmentation for both DL models and manual annotators relative to the consensus of the three annotators. This analysis provided an important measure of clinical reliability of segmentation across varying levels of error tolerance.

The $M_{P}^{CT}$ and $M_{P}^{MRI}$ segmentation models performed ably compared with human annotators, with good agreement between model and human annotations. Both models achieved high DSCs, ranging from 0.83 to 0.86, indicating good agreement between the predicted segmentations and the human annotator segmentations and their consensus segmentation. $M_{P}^{CT}$ model also outperformed the adult liver tumor segmentation model ($M_{A}^{CT}$) across all annotator comparisons, underscoring its superior consistency and reliability for pediatric cases. To our knowledge, this work is the first to demonstrate that liver tumor segmentation models trained on adult data do not generalize well to the pediatric population. These results are consistent with other work (19,21,32,33) showing that models trained on adult data do not perform at the same level when a similar task is performed using pediatric data. Specific to liver tumors, these reasons likely include the substantial differences in patient size, liver size, the average size of the liver tumor compared with the liver parenchyma, and the tumor imaging characteristics, including how hepatoblastoma tends to have a heterogeneous appearance with variable internal composition that can include calcification, hemorrhage, or small cystic spaces (17,34). These findings, along with evidence from other studies, emphasize the need for developing models specifically tailored to pediatric patients to achieve higher accuracy and better performance.

Our pediatric segmentation models also compare favorably with existing liver tumor segmentation models developed for adult patients. In earlier work, CC-DenseUNet (35) was proposed for CT-based liver tumor segmentation, achieving a DSC of 0.74. Another work using an attention-based model (36) for multiphase CT images reported a DSC of approximately 0.78. Arulappan et al (37) developed an asymmetric dilated convolutional encoder-decoder network for tumor segmentation in adult CT images, reaching an average DSC of 0.76. More recently, UNet++ was applied for liver tumor segmentation at MRI, yielding a lower DSC of 0.61 (38). Wesdorp et al (39) achieved a DSC of 0.86 for colorectal liver metastasis segmentation using an externally validated CT dataset. MULLET, a transformer-based model designed for multiphase contrast-enhanced CT, demonstrated comparable segmentation performance (DSC ≈ 0.78) and has been successfully deployed in clinical settings. Most recently, an AI-assisted platform (40) for hepatocellular carcinoma detection in CT achieved a DSC of 0.88 in an external clinical validation study. Although adult-trained models span a variety of liver tumor types and imaging modalities, our pediatric-specific models for hepatoblastoma achieved DSCs of 0.86 with both CT and MRI. This performance is comparable to, and in some cases exceeds, adult-focused approaches, despite the added challenge of working with a more heterogeneous and underrepresented pediatric tumor type. These results underscore our models’ ability to handle the unique complexities of pediatric liver tumors more effectively.

Our study also sheds light on the comparisons between novice and expert human annotators generating pediatric liver tumor segmentations. The results indicate that novice annotator (R_3_) segmentations had good agreement with those of expert annotators (R_1_ and R_2_) with DSCs of 0.88 for both CT and MRI segmentation tasks. However, the novice annotator showed lower performance than expert annotators when evaluated at very low (e < 1%) and low (e < 5%) error tolerance thresholds but had similar performance to expert annotators at moderate (e < 10%) and high (e < 15%) error tolerance thresholds. To our knowledge, this study provides one of the first comparative analyses of novice versus expert performance in hepatoblastoma segmentation. The data suggest that novice annotators do not perform at the same level as experts (especially at high precision tasks; ie, low error tolerance) and would likely benefit from input from expert’s oversight by those with subject matter expertise.

The VPE analyses highlight the inherent variability between the way human annotators and AI models define tumor boundaries. The expert annotators had slightly higher overall accuracy than the novice annotator and the $M_{P}^{CT}$ and $M_{P}^{MRI}$ models. This finding likely reflects the extensive experience of expert annotators and the refined techniques necessary to determine the most likely tumor margin—a task that is often complex. Not surprisingly, at higher error tolerance threshold, the distinctions between the preciseness of segmentations became less noticeable. This is because a larger error tolerance threshold (eg, 10%) will treat both small (eg, e = 1%) and large errors (eg, e = 9%) as acceptable, making it harder to see the differences in preciseness.

The annotators in our study showed tendencies toward both oversegmentation and undersegmentation, with inconsistencies observed among annotators and for the same annotator across different modalities (Table 5). Specifically, R_1_ and R_2_ exhibited a mix of undersegmentation and oversegmentation across modalities, although R_3_ consistently leaned toward oversegmentation, with no significant bias (*P* > .05). This result is not surprising and highlights the inherent variability and complexity in defining tumor boundaries. In contrast, the DL ($M_{P}^{CT}$ and $M_{P}^{MRI}$) segmentation models demonstrated a tendency toward undersegmentation in our tests, with the difference being significant for $M_{P}^{CT}$. Although there are general benefits and drawbacks to both over- and undersegmentation depending on the specific task, models that undersegment may have the benefit of increased likelihood of including only the true tumor within the defined region of interest, which may benefit AI analyses (eg, hepatoblastoma subtype identification). However, undersegmentation may come at the cost of the exclusion of tumor regions, some of which may represent the most active or aggressive portions of the tumor (41). This exclusion is particularly concerning when considering growth at the tumor periphery, which may impact subsequent radiomics-based analysis. Undersegmentation also results in an underestimation of the true tumor volume, which may be of consequence if such a metric is used to longitudinally track tumors and monitor response to therapy.

The spatial distribution of uncertainty observed in entropy analysis suggests that both DL models ($M_{P}^{CT}$ and $M_{P}^{MRI}$) exhibited higher confidence in the tumor cores and relatively higher uncertainty in boundary regions, where segmentation ambiguity is expected. These boundary areas often align with regions of known interobserver variability, reinforcing the interpretability and reliability of the model’s uncertainty output in clinical settings.

The ability of our pediatric-specific DL models to accurately segment hepatoblastoma carries meaningful clinical value. Accurate tumor delineation is essential for estimating tumor volume, which informs surgical planning, and enables precise tracking of tumor response during and after treatment. Reliable segmentation also facilitates radiomics analyses by providing consistent regions of interest for quantitative feature extraction, potentially aiding in subtype classification and prognostication (7,8). By reducing manual segmentation burden and interobserver variability, these models can streamline workflow in clinical and research settings, enhance reproducibility, and support more standardized care across institutions.

For real-world use, the proposed pediatric-specific segmentation models can be integrated into clinical workflows through picture archiving and communication systems or as part of dedicated AI workstations. These models can automatically generate tumor segmentations shortly after image acquisition, allowing radiologists to review and adjust results, thus reducing manual segmentation workload and saving time. Additionally, the outputs can serve as input for radiomics pipelines or tumor response monitoring tools, enabling early and objective decision-making. Successful clinical integration will require external validation across institutions, additional trial datasets, and deployment within secure, regulatory-compliant environments that support seamless interaction with existing imaging infrastructure.

Despite the promising results, our study had limitations. First, hepatoblastoma is a rare tumor. Our training and validation datasets are drawn from the Children’s Oncology Group AHEP0731 trial (NCT00980460), the largest and most diverse hepatoblastoma imaging dataset currently available, providing the best possible data foundation for constructing and validating DL models. As such, we derived data from one of the largest prospective studies ever performed, but our study includes only 104 CT and 123 MRI scans, a relatively small dataset for AI model construction. Second, the data are heterogeneous. The imaging studies included in the trial were performed at 105 unique imaging centers, each using their own institutional imaging protocols. Although this heterogeneity may impact the performance of the model, we believe that it also enhances the generalizability of our results. Third, the data used to train the models are now 6–16 years old, with the first scans in our dataset acquired in 2008 and the most recent acquired in 2018. It is not clear how advances in scanner hardware, acquisition techniques, and image reconstruction models could impact model performance. Thus, our AI segmentation models should be validated using more recently acquired scans. Fourth, the focus of our study on hepatoblastoma may limit model generalizability to other pediatric or adult liver tumors. Although the segmentation results are promising, their impact on subsequent radiomics analysis and the ability to predict disease outcomes remains unclear.

Future work should focus on external validation of these models using more recent clinical trial imaging data to assess performance on modern scanners and protocols. Broadening the scope to include segmentation of other pediatric liver tumors, such as hepatocellular carcinoma, fibrolamellar carcinoma, or metastatic lesions, could increase clinical utility. Additionally, it would be informative to explore how DL models, through guided learning with real-time feedback, can help novice annotators gain experience. In parallel, it is important to investigate how segmentation accuracy impacts radiomic feature extraction and predictive modeling.

In conclusion, we demonstrated that pediatric-derived AI models can segment hepatoblastoma using contrast-enhanced CT and T2-weighted fat-suppressed MRI with accuracy approaching near-human levels. The $M_{P}^{CT}$ model outperformed the adult liver tumor segmentation model $M_{A}^{CT}$, underscoring its superior consistency and reliability for pediatric cases. This study enables the integration of these segmentation models into clinical workflows, increasing the accuracy and efficiency of hepatoblastoma tumor delineation. By providing reliable segmentation, these models potentially support more precise treatment planning and monitoring and introduce opportunities for further assessment of more recent liver tumor trial data. Additionally, the reduction in manual segmentation efforts and interannotator variability facilitates streamlined radiologic assessments, empowering clinicians to make faster, data-driven decisions. Our work should serve as a foundation for the application of the current CT and MRI segmentation models to datasets containing patients with newer imaging studies and pediatric patients with other types of liver tumors. Our findings can aid in the development of new workflows and tools to quantitatively assess hepatoblastoma. Finally, our study findings emphasize the importance of developing and validating pediatric-specific DL models specifically tailored for pediatric populations.

## Supplemental Files

Table S1, Figures S1-S2

Conflicts of Interest
