# Supplementary material for: Pediatric Personalized Deep Learning Models for Segmentation of Hepatoblastoma at CT and MRI
Source: Radiol Imaging Cancer. 2026 Feb 20;8(2):e250041. doi: 10.1148/rycan.250041 (PMC13036680; doi:10.1148/rycan.250041)
Supplement: Table S1, Figures S1-S2 [file rycan250041suppa1.pdf]

Supplemental materials undergo peer review but are not copyedited, and may include typographical errors.

©RSNA, 2026

10.1148/rycan.250041

Table S1 Inference-time ablation results demonstrating the impact of omitting preprocessing steps on segmentation performance (DSC) for CT and MRI validation cohorts

Modality	Preprocessing Step Omitted	Baseline DSC	Ablated DSC	ΔDSC (%)
CT	HU normalization	0.86	0.83	–3.49%
MRI	N4 biasfield correction + histogram standardization	0.86	0.77	–10.47%

Baseline DSC denotes performance with the full pipeline; Ablated DSC denotes performance after removal of the specified step(s); ΔDSC (%) represents the relative change.

Supplemental materials undergo peer review but are not copyedited, and may include typographical errors.

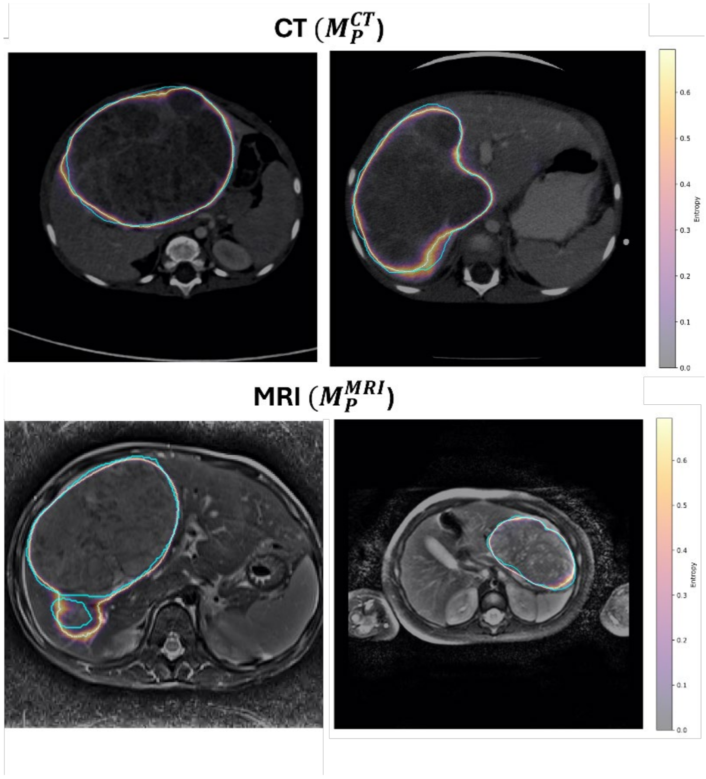

Figure S1: Representative uncertainty overlays for pediatric hepatoblastoma segmentations on CT (top row) and MRI (bottom row) cases. Entropy maps, derived from the softmax output, are shown as colored overlays indicating voxel-wise model uncertainty (colormap: low = gray, high = yellow). Cyan contours represent the consensus reference segmentation, while white contours indicate model predictions. Uncertainty is predominantly concentrated along tumor boundaries—regions typically associated with anatomical ambiguity and segmentation variability.

Supplemental materials undergo peer review but are not copyedited, and may include typographical errors.

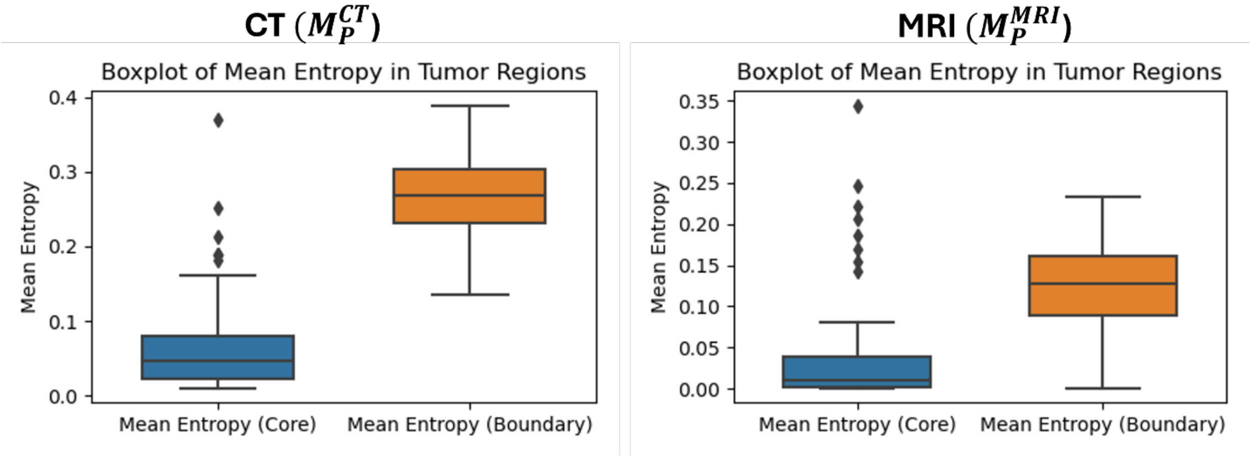

Figure S2: Boxplots of mean voxel-wise entropy within tumor regions for CT (left) and MRI (right). Entropy was computed from the softmax output at inference time. The tumor core was defined as the eroded interior of the predicted tumor mask using a 2D disk-shaped structuring element (radius = 10 pixels) applied slice-wise, while the boundary was defined as the outer rim of the tumor (i.e., the difference between the original mask and its eroded version). Across both modalities, mean entropy was significantly higher in the boundary regions, reflecting greater model uncertainty in anatomically ambiguous zones.
