# Supplementary material for: Pediatric Personalized Deep Learning Models for Segmentation of Hepatoblastoma at CT and MRI
Source: Radiol Imaging Cancer. 2026 Feb 20;8(2):e250041. doi: 10.1148/rycan.250041 (PMC13036680; doi:10.1148/rycan.250041)
Supplement: Conflicts of Interest [file rycan250041coi.zip › 538923899_20987064_1753786598145.pdf]

ICMJE DISCLOSURE FORM

Instructions

In the interest of transparency, we ask you to disclose all employment/relationships/activities/interests listed below that are related to the content of your manuscript. “Related” means any relationship with for-profit or not-for-profit third parties whose interests may be affected by the content of the manuscript. Disclosure represents a commitment to transparency and does not necessarily indicate a bias. If you are in doubt about whether to list an employment/relationship/activity/interest, it is preferable that you do so.

The following questions apply to your employment/relationships/activities/interests as they relate to the **current manuscript only**. Each author is required to submit a separate form and is responsible for the accuracy and completeness of the submitted information.

Your employment/relationships/activities/interests should be **defined broadly**. For example, if your manuscript pertains to the epidemiology of hypertension, you should declare all relationships with manufacturers of antihypertensive medication, even if that medication is not mentioned in the manuscript.

Date: 29-Jul-2025

req First Name: Anant

req Last Name: Madabhushi

Manuscript Title: Pediatric Personalized Deep Learning Models for Segmentation of Hepatoblastoma on CT and MRI

Manuscript number: RYCAN-25-0041.R1

In item #1 below, report all support for the work reported in this manuscript without time limit. For all other items, the time frame for disclosure is the past 36 months. Note: All items #1 through #13 must indicate none (by checking the box next to None) or include relevant disclosure information in the text boxes. Blank rows will cause the form to be sent back for completion.

	**Name all entities with whom you have this relationship or check the box next to None**	**Specifications/Comments (e.g., if payments were made to you or to your institution)**
**Time frame: Since the initial planning of the work**		
1. All support for the present manuscript (e.g., funding, provision of study materials, medical writing, article processing charges, etc.)<br>**No time limit for this item.**	☐	None

	Research reported in this publication was supported by ARPA-H under contract number D25AC00140, National Cancer Institute under award numbers R01CA268287A1, U01CA269181, R01CA26820701A1, R01CA249992-01A1, R01CA202752-01A1, R01CA208236-01A1, R01CA216579-01A1, R01CA220581-01A1, R01CA257612-01A1, 1U01CA239055-01, 1U01CA248226-01, 1U54CA254566-01, National Heart, Lung and Blood Institute 1R01HL15127701A1, R01HL15807101A1, VA Merit Review Award IBX004121A from the United States Department of Veterans Affairs Biomedical Laboratory Research and Development Service the Office of the Assistant Secretary of Defense for Health Affairs, through the Prostate Cancer Research Program (W81XWH-20-1-0851) and sponsored research agreements from Bristol Myers-Squibb, and Astrazeneca.<br><br>The content is solely the responsibility of the authors and does not necessarily represent the official views of the National Institutes of Health, the U.S. Department of Veterans Affairs, the Department of Defense, or the United States Government.	Institution
**Time frame: past 36 months**		
2. Grants or contracts from any entity (if not indicated in item #1 above).	✓	None
3. Royalties or licenses	✓	None
4. Consulting fees	☐	None
	Dr Madabhushi is a Research Career Scientist at the Atlanta Veterans Affairs Medical Center. Dr. Madabhushi is an equity holder in Picture Health, Elucid	

	Bioimaging, and Inspirata Inc. Currently he serves on the advisory board of Picture Health. He currently consults for Takeda Inc. He also has sponsored research agreements with AstraZeneca and Bristol Myers-Squibb. His technology has been licensed to Picture Health and Elucid Bioimaging. He is also involved in 1 R01 grant with Inspirata Inc.	
5. Payment or honoraria for lectures, presentations, speakers bureaus, manuscript writing or educational events	✓	None
6. Payment for expert testimony	✓	None
7. Support for attending meetings and/or travel	✓	None
8. Patents planned, issued or pending	☐	None
	"Feature driven local cell graph (FeDeG): Predicting overall survival in early stage lung cancer"<br>Anant Madabhushi, Cheng Lu<br>USSN: 11,455,718 – September 27th, 2022<br><br>"Predicting Recurrence in Lung Cancer with Phenotyping Tumor Infiltrating Lymphocytes (PhenoTIL) on Hematoxylin and Eosin (H&E) Tissue Images"<br>Anant Madabhushi, German Corredor Prada, Cristian Barrera, Eduardo Romero<br>USSN: 11,461,891 – October 4th, 2022<br><br>"Distinguishing minimally invasive carcinoma and adenocarcinoma in situ from invasive adenocarcinoma with intratumoral and peri-tumoral textural features"<br>Anant Madabhushi, Kaustav Bera, Pranjal Vaidya<br>USSN: 11,464,473 – October	

	11th, 2022<br><br>"Prognosis of Prostate Cancer with Computerized Histomorphometric Features of Tumor Morphology from Routine Hematoxylin and Eosin Slides"<br>Anant Madabhushi, Patrick Leo, Andrew Janowczyk, Kaustav Bera<br>USSN: 11,494,900 – November 8th, 2022<br><br>"Prediction of risk of post-ablation atrial fibrillation based on radiographic features of pulmonary vein morphology from chest imaging"<br>Anant Madabhushi, Michael LaBarbera, Thomas Atta-Fosu, Mina Chung<br>USSN: 11,540,796 – January 3rd, 2023<br><br>"Radiographic-Deformation and Textural Heterogeneity (R-Depth): An Integrated Descriptor for Brain Tumor Prognosis"<br>Pallavi Tiwari, Anant Madabhushi, Prateek Prasanna<br>USSN: 11,555,877 – January 17th, 2023<br><br>"Predicting Recurrence and Overall Survival Using Radiomic Features Correlated with PD-L1 Expression in Early Stage Non-Small Cell Lung Cancer (ES-NSCLC)"<br>Anant Madabhushi, Pranjal Vaidya, Kaustav Bera, Prateek Prasanna, Vamsidhar Velcheti<br>USSN: 11,574,404 – February 7th, 2023<br><br>"Fractal Analysis of Left Atrium to Predict Atrial Fibrillation Recurrence"<br>Anant Madabhushi, Marjan Firouznia, Mina K Chung, Albert Feeny<br>USSN: 11,576,640 – February 14th, 2023<br><br>"Predicting tumor prognoses based on a combination of radiomic and clinico-pathological features"<br>Pranjal Vaidya, Kaustav Bera, Anant Madabhushi	

	USSN: 11,610,304 – March 21st, 2023<br><br>“Deep Learning-based Multi-site, Multi-primitive segmentation for nephropathology using renal biopsy whole slide images”<br>Anant Madabhushi, Catherine Jayapandian, Yijiang Chen, Andrew Janowczyk, John Sedor, Laura Barisoni<br>USSN: 11,645,753 – May 9th, 2023<br><br>“Combination of Radiomic and Pathomic Features in the Prediction of Prognoses for Tumors”<br>Pranjal Vaidya, Anant Madabhushi, Kaustav Bera<br>USSN: 11,676,703 – June 13th, 2023<br><br>“Structural Rectal Atlas Deformation Features for Characterizing Intra-Wall and Peri-Wall Chemoradiation Response on Magnetic Resonance Imaging (MRI)”<br>Anant Madabhushi, Jacob Antunes, Zhouping Wei, Pallavi Tiwari, Satish E. Viswanath, Charlems Alvarez Jimenz<br>USSN: 11,798,179 – October 24th, 2023<br><br>“Disease Characterization and Response Estimation Through Spatially-Invoked Radiomics and Deep Learning”<br>Anant Madabhushi, Nathaniel Braman, Jeff Eben<br>USSN: 11,810,292 – November 7th, 2023<br><br>“Specialized Computer-Aided Diagnosis and Disease Characterization with a Multi-Focal Ensemble of Convolutional Neural Networks”<br>Anant Madabhushi, Nathaniel Braman, Tristan Maidment, Yijiang Chen<br>USSN: 11,817,204 – November 14th, 2023<br><br>“Image analysis method, estimating device, estimating system, and storage medium”	

	Kazuaki Nakane, Chaoyang Yan, Xiangxue Wang, Yao Fu, Haoda Lu, Xiangshan Fan, Michael D. Feldman, Anant Madabhushi, Jun Xu<br>USSN: 11,861,836 – January 2nd, 2024<br><br>“Tumor Characterization and Outcome Prediction through Quantitative Measurements of Tumor-Associated Vasculature”<br>Anant Madabhushi, Nathaniel Braman<br>USSN: 11,896,349 – February 13th, 2024<br><br>“Predicting Overall Survival in Early Stage Lung Cancer with Feature Driven Local Cell Graphs (FEDEG)”<br>Anant Madabhushi, Cheng Lu<br>USSN: 11,922,625 – March 5th, 2024<br><br>“Predicting Neo-Adjuvant Chemotherapy Response From Pre-Treatment Breast Magnetic Resonance Imaging Using Artificial Intelligence and HER2 Status”<br>Anant Madabhushi, Nathaniel Braman, Kavya Ravichandran, Andrew Janowczyk<br>USSN: 11,922,625 – May 14th, 2024<br><br>“Population-Specific Prediction of Prostate Cancer Recurrence based on Stromal Morphology Features”<br>Anant Madabhushi, Hersh Bhargava, Patrick Leo, Priti Lal<br>USSN: 12,008,747 – June 11th, 2024<br><br>“Distinguishing Colon Cancer Stages Based on Computationally Derived Morphological Features of Cancer Nuclei”<br>Anant Madabhushi, Neeraj Kumar, Joseph E Willis<br>USSN: 12,014,488 – June 18th, 2024<br><br>“Computational Features of Tumor-Infiltrating Lymphocyte (TIL) Architecture”<br>Anant Madabhushi, Sepideh	

	Azarianpour, Mahdi Haider<br>USSN: 12,073,560 – August 27th, 2024<br><br>"The Combination of features from biopsies and scans to predict prognosis in SCLC"<br>Anant Madabhushi, Cristian Barrera, Mohammadhadi Khorrami, Prantesh Jain, Afshin Dowlati<br>USSN: 12,159,403 – December 3rd, 2024<br><br>"Intra-Perinodular Textural Transition (IPRIS): A Three Dimensional (3D) Descriptor for Nodule Diagnosis on Lung Computed Tomography (CT) Images"<br>Mehdi Alilou, Anant Madabhushi<br>US Reissued Patent: RE50,286 E – January 28th, 2025	
9. Participation on a Data Safety Monitoring Board or Advisory Board	✓	None
10. Leadership or fiduciary role in other board, society, committee or advocacy group, paid or unpaid	☐	None
	Dr Madabhushi serves as Chief Scientific Officer for Picture Health.	
11. Stock or stock options	☐	None
	Dr Madabhushi has an equity stake in Picture Health, Elucid Bioimaging and Inspirata Inc.	
12. Receipt of equipment, materials, drugs, medical writing, gifts or other services	✓	None
13. Other financial or non-financial interests	✓	None

req **Please check the box next to the following statement to indicate your agreement:**

✓ **I certify that I have answered every question and all the information is complete and accurate.**

This is a reprint of the ICMJE Recommendations for the Conduct, Reporting, Editing and Publication of Scholarly Work in Medical Journals. RSNA prepared this reprint. The ICMJE has not endorsed nor approved the contents of this reprint. The official version of the Recommendations for the Conduct, Reporting, Editing and Publication of Scholarly Work in Medical Journals is located at www.ICMJE.org. Users should cite this official version when citing the document.
